# Supplementary material for: Predictive Value of Hepatitis B Core-Related Antigen for Multiple Recurrence Outcomes After Treatment Cessation in Chronic Hepatitis B: A Meta-Analysis Study
Source: Viruses. 2025 Jun 30;17(7):929. doi: 10.3390/v17070929 (PMC12299212; doi:10.3390/v17070929)
Supplement: Supplementary file 1 [file viruses-17-00929-s001.zip › Table S1.pdf]

Table S1:Characteristics of the included studies.

| Author and year | Country | Study type | sample year | No. of patients (F/M) | Baseline HBeAg status （+/total patients） | Baseline HBsAg （log10 U/mL） | Baseline HBcrAg （log10 U/mL） | The average age （years） | Detection Time / Prediction Time / Outcome | Cut-off value （log10 U/mL） | AUC（95% CI） [Sen/Sep] | Method of measurement | Inclusion Criteria | Exclusion Criteria |
|---|---|---|---|---|---|---|---|---|---|---|---|---|---|---|
| Kaewdech, Apichat et al. （2020） | Thailand | prospective, observational study | 2018.02-2019.08 | 33/59 | 20/92 | 2.96 (2.40-3.43) | 3.20 (<3.00-3.90) | 55.0 (50.0-63.0) | EOT HBcrAg prediction / EOT 48w / Relapse | 3 | 0.773 （0.677-0.869） [90.3%/50.8%] | CLEIA | ①18 years of age or older and had been treated with long-term NA；<br>②All patients fulfilled the APASL consensus statement | ①Patients with advanced fibrosis or cirrhosis；<br>②Patients with active hepatocellular carcinoma or other malignancies；<br>③Patients with co-infection with hepatitis C virus or human immunodeficiency virus；<br>④Patients undergoing concomitant treatment with immunosuppressive agents；<br>⑤Patients with other chronic liver disease；<br>⑥Pregnancy or lactating women；<br>⑦Patients refused to participate in the study. |
| Liu, Ruyu et al. （2023） | China | prospective study | 2019.01-2022.04 | 172/0 | 172/172 | >3 | <3 | 26.49 ± 3.77 | Postpartum 12-week (12w) EOT HBcrAg prediction / Postpartum 12w / Hepatitis B acute flare | 0.275(OD value） | 0.84（0.78-0.91） [86.5%/67.5%] | ELISA | ①Pregnant chronic HBV infected women who underwent short-course antiviral therapy with TDF ；<br>②Pregnant patients were judged as chronic HBV infection and to be in the immune-tolerant phase;<br>③Patients had normal alanine aminotransferase at 24 weeks gestation. | ①Coinfection with HIV or hepatitis C, D virus；<br>②Hepatocellular carcinoma patients；<br>③Treatment with liverprotecting drugs patients；<br>④Immunosuppressive treatment ;<br>⑤Liver disease caused by reasons other than HBV infection. |
| Wang, Fa-Da et al. （2022） | China | retrospective study | 2020.06-2021.01 | 32/32 | 36/64 | HBeAg(+): 3.41 (2.97–2.47)<br>HBeAg(-): 3.05 (2.77–3.42) | HBeAg(+): 3.34 ± 0.10<br>HBeAg(-): 3.08 ± 0.09 | HBeAg(+): 47.28 ± 6.86<br>HBeAg(-): 47.39 ± 6.45 | EOT HBcrAg prediction / Within 1 year after treatment cessation / Relapse | 3.3 | 0.817 [78%/77%] | CLEIA | ①Age ≥18 years, HBsAg positivity and undetectable HBV DNA at the time of NAs discontinuation;<br>②HBeAg-positive and HBeAg-negative patients who meet the AsiaPacific guidelines of NAs discontinuation criteria.<br>③HBeAg positive patients were required to achieve HBeAg seroconversion and undetectable HBV DNA for at least 12 months of consolidation therapy;<br>④HBeAg negative patients were required to achieve undetectable HBV DNA and then at least 18 months of intensive treatment. | ①Coinfection with hepatitis C virus, hepatitis D virus, or human immunodeficiency virus；<br>②Immunocompromised status or malignancy；<br>③Autoimmune liver disease, alcohol abuse, previous history of liver transplantation, and other severe or active disease；<br>④History of decompensated liver disease or presence of decompensated cirrhosis. |

| | | | | | | | | | | | | | | |
|---|---|---|---|---|---|---|---|---|---|---|---|---|---|---|
| Matsumoto, Akihiro et al.（2007） | Japan | retrospective study | [N/A] | 14/20 | 16/34 | positive | 6.3（5.0-7.7） | 47（35-59） | EOT HBcrAg Prediction / Within 1 Year After Treatment Cessation / Acute Hepatitis B Flare | 4.1-4.6 | 0.764 [80%/80%] | CLEIA | Patients with chronic hepatitis B who were treated with lamivudine for at least 6 months；<br>*The patients were selected retrospectively from five medical institutions in Japan. | [N/A] |
| Fan, Rong et al.（2020） | China | prospective study | [N/A] | 35/92 | 127/127 | 3.3 (2.9 - 3.7) | 4.3 (4.0 - 4.7) | 30 (25 - 35) | EO HBcrAg Prediction / Within 4 Years After EOT / Relapse | 4 | 0.621 [N/A] | CLEIA | Adult patients with HBV DNA ≥ 20 000 IU/mL, alanine aminotransferase (ALT) 2–10 times the upper limit of normal (ULN), and compensated naive HBeAg-positive CHB received telbivudinebased antiviral treatment. | [N/A] |
| | | | | 13/46 | 59/59 | 2.6 (1.4 - 3.0) | 3.9 (3.3 - 4.5) | 36 (30 - 43) | EO HBcrAg Prediction / Within 4 Years After EOT / Relapse | 4 | 0.798 [N/A] | CLEIA | The patients in the validation cohort were enrolled from another prospective trial conducted in Nanfang Hospital (Guangzhou, China). | |
| Liu, Yiqi et al.（2023） | China | retrospective study | 2010.06-2021.12 | 23/30 | 53 | positive | 4.27 ± 1.99 | 53.2 ± 12.0 | Pre-chemotherapy HBcrAg Prediction / 48 Weeks Post-chemotherapy / HBV Reactivation | 4.668 | 0.689 [N/A] | CLEIA | ①Patients were HBsAg−positive, or HBsAg−negative but anti-HBc-positive;<br>②Patients had a confirmed diagnosis of diffuse large B-cell lymphoma (DLBCL) from biopsy results;<br>③Patients received at least four cycles of immunochemotherapy<br>Group B：HbsAg+ | ①Patients had involvement of the central nervous system;<br>②Patients had a human immunodeficiency virus or other hepatitis virus coinfection.<br>③Less than four chemotherapy cycles，incomplete baseline data or coinfected with HCV，etc. |
| | | | | 29/38 | 67 | negative | 2.67 ± 0.54 | 62.5 ± 11.5 | Pre-chemotherapy HBcrAg Prediction / 48 Weeks Post-chemotherapy / HBV Reactivation | 2.54 | 0.605 [N/A] | CLEIA | ①Patients were HBsAg−positive, or HBsAg−negative but anti-HBc-positive; ② patients had a confirmed diagnosis of diffuse large B-cell lymphoma (DLBCL) from biopsy results;<br>③Patients received at least four cycles of immunochemotherapy<br>Group C：HbsAg−/anti-HBc+ patients | |
| Huang, Da et al.（2024） | China | RCT | N/A | 14/66 | 33/80 | 2.797±0.425 | N/A | 35.4±9.8 | NAs EOT HBcrAg Prediction / During Interferon Conversion Therapy / HBV Reactivation | 5 | 0.755 [N/A] | CLEIA | ①CHB patients aged 18–65 years;<br>②Received NA treatment for 1–5 years;<br>③Serum HBV DNA ≤1000 copies/mL and HBsAg≤ 3000 IU/mL. | ①Receiving IFN or systemic antiviral therapy within the previous 6 months;<br>②Coinfection with the human immunodeficiency virus, hepatitis C or D virus;<br>③Presence of decompensated liver disease;<br>④Pregnancy or lactation; |

| | | | | | | | | | | | | | | |
|---|---|---|---|---|---|---|---|---|---|---|---|---|---|---|
| | | | | | | | | | Baseline HBcrAg Prediction / During Interferon Transition Therapy / HBV Reactivation | N/A | 0.713 [N/A] | | | ⑤Having any other contraindication for IFN therapy. |
| Zhou,Fang et al.(2024) | China | prospective study | 2020.06-2021.06 | Relapse group：16/22<br>Non-relapse group：36/34 | Not mentioned/108 | 2.82±0.08 | 6.075±1.52 | 42.83±9.63 | EOT HBcrAg Prediction / 1 Year Later / Post-Treatment Relapse | N/A | 0.783（0.650-0.884）[68.42%/82.68%] | CLEIA | ①Met the diagnostic criteria for CHB and had received standardized treatment;<br>②Had completed sufficient treatment course and met the discontinuation criteria: post-treatment HBV DNA load below the detection limit (<100 IU/mL) with at least 3 follow-up tests at 6-month intervals all showing undetectable levels, concurrent with hepatitis B virus surface antigen (HBsAg) <3000 IU/mL;<br>③Aged between 18-70 years;<br>④Had complete clinical data and could cooperate throughout the entire study process. | ①Patients with concurrent alcoholic liver disease, non-alcoholic fatty liver disease, autoimmune hepatitis, primary liver cancer, or other hepatic disorders;<br>②Decompensated liver cirrhosis;<br>③Women who were pregnant or breastfeeding;<br>④Co-infection with hepatitis C virus (HCV), human immunodeficiency virus (HIV), Mycobacterium tuberculosis, or other viral/bacterial infections;<br>⑤Patients with psychiatric disorders affecting compliance;<br>⑥Patients with malignancies, autoimmune diseases, or severe cardiovascular/cerebrovascular diseases;<br>⑦Cases with primary or secondary drug resistance. |
| Liu,Yaodan et al.(2024) | China | prospective observational study | 2020.01-2022.10 | 198/0 | 198/198 | Postpartum Hepatitis Non-flare Group: 3.66 (3.20-4.01)<br>Postpartum Hepatitis Flare Group: 4.05 (3.70-4.24) | Postpartum Hepatitis Non-flare Group: 6.98 (6.77-7.18)<br>Postpartum Hepatitis Flare Group: 7.35 (7.31-7.52) | Postpartum Hepatitis Non-flare Group: 30.89 ± 6.13<br>Postpartum Hepatitis Flare Group: 29.88 ± 4.55 | HBcrAg at Antiviral Treatment Initiation / 48 Weeks Postpartum/ alt flare | N/A | 0.713 [N/A] | ELISA | ①Pregnant women meeting the criteria for antiviral therapy to prevent mother-to-child transmission as specified in both the Chronic Hepatitis B Prevention and Treatment Guidelines (2019 Edition) and the *Clinical Guidelines for Prevention of Hepatitis B Virus Mother-to-Child Transmission (2020 Edition)*.<br>②Other cases receiving antiviral prophylaxis during pregnancy. | ①Patients receiving antiviral therapy for active chronic hepatitis B infection<br>②Patients with:<br>Coinfection with other hepatotropic viruses、HIV infection、Liver cirrhosis、Pregnancy-related liver diseases (e.g., intrahepatic cholestasis of pregnancy) |

| | | | | | | | | | | | | | | |
|---|---|---|---|---|---|---|---|---|---|---|---|---|---|---|
| Liao,Guichan.(2022) | China | prospective study | 2011.11-2018.12 | 27/95 | 122/122 | 2.55±1.11 | 3.80±0.83 | 34（29-40） | EOT HBcrAg Prediction / 5 Years Post-EOT / Relapse | 4.0 | 0.71（0.62-0.81）[87.1%/61.5%] | CLEIA | Stopping Criteria Based on 2012 APASL Guidelines:<br>① Pre-treatment HBeAg-positive status;<br>②Age 18-69 years (inclusive) for both male and female patients;<br>③Consolidation therapy after achieving:HBV DNA below the detection limit；ALT normalization；HBeAg seroconversion(Minimum 12 months of continued oral nucleos(t)ide analog therapy post-seroconversion);<br>④Undetectable HBV DNA (<20 IU/mL, measured by cobas® assay with internal standards) on ≥2 consecutive tests;<br>⑤Written informed consent obtained after full explanation of study procedures, with commitment to comply with research requirements. | ①Evidence of hepatic fibrosis or cirrhosis by: Liver ultrasound and/or Liver elastography (FibroScan) and/or Liver biopsy<br>②Coinfection with other viruses, including: Hepatitis C virus (HCV)；Hepatitis D virus (HDV)；Human immunodeficiency virus (HIV)<br>③History of hepatic decompensation, such as: Ascites；Gastroesophageal variceal bleeding；Hepatic encephalopathy<br>④Laboratory abnormalities: Total bilirubin (TBIL) ≥2× upper limit of normal (ULN) or Prothrombin time prolongation ≥3 seconds vs. ULN or Serum albumin (ALB) <32 g/L<br>⑤Suspicious hepatic nodules on imaging (ultrasound/CT/MRI) or AFP >20 ng/mL<br>⑥Concurrent severe/active comorbidities, including uncontrolled: Renal/cardiac/pulmonary/vascular/neurological/gastrointestinal diseases/Autoimmune liver diseases/Metabolic disorders/Immunodeficiency syndromes/Malignancies<br>⑦Planned immunosuppressive therapy<br>⑧Prior or planned liver transplantation<br>⑨Poor adherence or inability to comply with study protocols |
| Liu,Zhongwei.(2022) | China | prospective study | [N/A] | [N/A] | Not mentioned/ 41 | 2.07（1.315 - 2.808） | 3.58±0.75 | 42.09±8.13 | EOT HBcrAg Prediction / During Follow-up / HBsAg Recurrence | 3.8 | 0.793 [66.67%/78.12%] | CLEIA | Inclusion Criteria for HBeAg-Positive CHB Patients Receiving Outpatient Interferon Therapy:<br>①Age: 18–60 years (inclusive)<br>②Diagnostic Confirmation: Persistent HBsAg positivity (>6 months)；Baseline virological markers: HBsAg <2000 IU/mL、HBV DNA <500 IU/mL、HBeAg-positive with quantitative level <50 COI；Biochemical marker: ALT ≤2× upper limit of normal (ULN)<br>③Treatment Status: Either currently receiving nucleos(t)ide analog therapy or treatment-naïve<br>④Informed Consent: Patients and their families fully understand and voluntarily sign informed consent documents. | ①Contraindications per clinician assessment: Autoimmune diseases；Severe psychiatric disorders；Other interferon contraindications as determined by treating physician<br>②Known hypersensitivity: Allergy to interferon or any component of the formulation<br>③Viral coinfections: Other viral hepatitis infections (HCV, HDV, etc.)；Human immunodeficiency virus (HIV) infection<br>④Advanced liver disease: Severe cirrhosis (Child-Pugh score >6)；Hepatocellular carcinoma (HCC)<br>⑤Recent immunomodulatory therapy: Any immunomodulatory treatment within the past 6 months<br>⑥Pregnancy-related exclusions: Current pregnancy；Pregnancy planned within next 2 years |

| | | | | | | | | | | | | | | |
|---|---|---|---|---|---|---|---|---|---|---|---|---|---|---|
| Aihaiti et al.(2020) | China | prospective study | 2014.08-2017.06 | 30/166 | Not mentioned/ 196 | N/A | 4.9（2-7.5） | 55（40-74） | Post-LT Day 3 HBcrAg Prediction / 3 Years Post-Transplant / HBV Recurrence | 4.5 | 0.818 （0.72-0.917） [76.19%/77.14%] | CLEIA | ①Meeting transplant indications: Fulfills standard criteria for liver transplantation；Has suitable donor availability<br>②Etiology confirmation: Diagnosis of hepatitis B virus (HBV)-associated liver disease<br>③Data completeness: Complete clinical records available<br>④Compliance: Demonstrated good medication adherence and follow-up compliance | Exclusion Criteria for Liver Transplant Recipients with HBV-related Liver Disease:<br>①Non-compliance with medication and follow-up: Inability to adhere to prescribed immunosuppressive/antiviral regimens during the perioperative and postoperative recovery periods；Failure to attend scheduled clinical follow-ups<br>②Early post-transplant mortality: Death due to acute graft rejection within the initial postoperative phase<br>③HBV-positive donor grafts: Recipients of liver grafts from HBsAg-positive donors<br>④Early re-transplantation: Patients requiring repeat liver transplantation within 1 year of initial procedure |
| Gao,Chang.(2019) | China | prospective study | 2003.01-2011.07 | 3/33 | 25/36 | 2.78 （2.02-3.32） | 3.85 （3.0-4.4） | 35.5 （29.25-41） | EOT 12-week HBcrAg Prediction / EOT 48-week / Relapse | 4.1 | 0.719 （0.536-0.902） [73.7%/76.9%] | CLEIA | ①Patient Eligibility: Adult patients (aged 18–69 years) willing to discontinue treatment, who understood and signed informed consent and met all study requirements.<br>②Treatment Requirements: HBeAg-positive at baseline: Must have received continuous oral NAs therapy until: HBV DNA <LLOQ (lower limit of quantification)、ALT normalization、Required ≥12 months of consolidation therapy after HBeAg seroconversion；HBeAg-negative at baseline: Required ≥18 months of consolidation therapy after achieving: HBV DNA <LLOQ ALT normalization.<br>③Virological Confirmation at Discontinuation:HBV DNA <20 IU/mL (confirmed via Cobas TaqMan assay, Roche Molecular Systems, Pleasanton, CA, USA).<br>④Liver Disease Status:Compensated liver disease at baseline, with no evidence of cirrhosis.<br>⑤Laboratory Parameters at Screening: WBC >$3.5\times10^9$/L；PLT >$100\times10^9$/L；AFP <20 ng/mL；ALT/AST ratio within normal limits；HBV DNA below | ①Poor compliance as judged by investigators or inability to adhere to study requirements.<br>②Presence of decompensated liver disease during previous treatment, including but not limited to: Total bilirubin (TBiL) >2×ULN；Prothrombin time prolonged by 2-3 seconds compared to ULN；Serum albumin (ALB) <32g/L；History of hepatic decompensation (e.g., ascites, variceal bleeding, or hepatic encephalopathy)；Suspicious nodules detected by ultrasound or radiological examination or AFP>20ng/mL<br>③Liver biopsy (prior to treatment cessation) showing significant bridging fibrosis (>S3), or FibroScan (prior to treatment cessation) showing significant fibrosis or cirrhosis.<br>④Coinfection with other viruses including hepatitis C virus (HCV), hepatitis D virus (HDV), or human immunodeficiency virus (HIV), or presence of autoimmune liver diseases.<br>⑤Concurrent active or severe diseases, including any clinically significant uncontrolled conditions affecting: Organs (kidneys, heart, lungs)；Vascular, neurological, or digestive systems；Metabolic disorders (e.g., diabetes, thyroid diseases, adrenal diseases)；Immunodeficiency disorders or malignancies |

| | | |
|---|---|---|
| | detection limit | ⑥Patients who have undergone liver transplantation or are scheduled for liver transplantation. |
