# Supplementary material for: Predictive Value of Hepatitis B Core-Related Antigen for Multiple Recurrence Outcomes After Treatment Cessation in Chronic Hepatitis B: A Meta-Analysis Study
Source: Viruses. 2025 Jun 30;17(7):929. doi: 10.3390/v17070929 (PMC12299212; doi:10.3390/v17070929)
Supplement: Supplementary file 1 [file viruses-17-00929-s001.zip › Table S2.pdf]

Table S2: Meta-Regression analysis of HBcrAg in predicting HBV recurrence outcomes: after exclusion of studies

Variable	No. of Studies	β(95%CI)	SE	p-value	Residual Heterogeneity (I^2_res, %)	Adjusted R^2 (%)
Age	11	0.005 (-0.015, 0.025)	0.0087	0.569	8.3	-8.4
Gender ratio	11	0.350 (-0.429, 1.129)	0.344	0.336	1	1.87
HBeAg+ rate	7	0.048 (-0.732, 0.827)	0.303	0.881	0	N/A
HBsAg level	8	0.046 (-0.153, 0.246)	0.082	0.591	0	N/A
HBcAb level	9	0.086 (-0.081, 0.252)	0.07	0.263	1.9	16
Publication year	11	-0.038 (-0.119, 0.044)	0.036	0.325	1.6	30.3
**Endpoint type (Ref: Relapse)**	11			(p = 0.259)	0	100
HBVr		-0.058 (-0.698, 0.581)	0.261	0.831		
VBT		0.357 (-0.436, 1.150)	0.324	0.313		
Acute exacerbation		0.044 (-0.568, 0.656)	0.25	0.865		
Other		0.488 (-0.089, 1.064)	0.235	0.084		
**Cut-off value (Ref: ≥4 log_{10} U/mL)**	11			(p = 0.607)	9.1	-40.5
3-4log_{10}IU/mL		0.138 (-0.555, 0.831)	0.293	0.652		
<3log_{10}IU/mL		-0.372 (-1.226, 0.482)	0.361	0.338		
≥0.275OD		-0.210 (-0.952, 0.531)	0.314	0.524		
**Detection time (Ref: Postoperative day 3)**	11			(p = 0.548)	0	17.7
EOT		-0.314 (-0.999, 0.370)	0.28	0.304		
Pre-chemotherapy		-0.540 (-1.338, 0.259)	0.326	0.149		
EOT 12w		-0.109 (-1.766, 1.548)	0.677	0.877		
Postpartum W12 HBcrAg		-0.483 (-1.314, 0.347)	0.34	0.204		

Variable	No. of Studies	β(95%CI)	SE	p-value	Residual Heterogeneity (I²_res, %)	Adjusted R² (%)
**Study quality (Ref: Moderate risk)**	11				16.9	-36.5
Low risk		-0.032 (-0.550, 0.486)	0.229	0.892		
**Study design (Ref: Retrospective)**	11			(p = 0.819)	20.8	-44.7
RCT		0.255 (-0.664, 1.174)	0.399	0.54		
Prospective		0.059 (-0.515, 0.633)	0.249	0.819		

Age: Represented by the median or mean age of participants in each study.HBsAg Level: Baseline HBsAg values (median or mean) from included studies, measured in log IU/mL.HBcrAg Level: Baseline HBcrAg values (median or mean), reported in log U/mL.

All tested covariates—including age, gender ratio, HBeAg-positive proportion, HBsAg/HBcrAg levels, publication year, endpoint definitions, cutoff values, detection timing, study design, and quality—**did not significantly modify** the predictive effect of HBcrAg (all $p > 0.05$). This suggests that HBcrAg's performance is consistent across diverse populations and methodologies and the variability in cutoff values (not covariates) primarily contributed to heterogeneity,as shown in subgroup analyses
