# Supplementary material for: Predictive Value of Hepatitis B Core-Related Antigen for Multiple Recurrence Outcomes After Treatment Cessation in Chronic Hepatitis B: A Meta-Analysis Study
Source: Viruses. 2025 Jun 30;17(7):929. doi: 10.3390/v17070929 (PMC12299212; doi:10.3390/v17070929)
Supplement: Supplementary file 1 [file viruses-17-00929-s001.zip › Table S3.pdf]

Table S3: Meta-Regression analysis of HBcrAg in predicting HBV recurrence outcomes: before exclusion of studies

Variable	No. of Studies	β(95%CI)	SE	p-value	Residual Heterogeneity (I²_res, %)	Adjusted R² (%)
Age	15	0.017(-0.017,0.051)	0.0158	0.301	83.9	2.73
Gender ratio	15	0.638(-0.681,1.956)	0.61	0.315	84.6	0.72
HBeAg+ rate	9	-0.293(-1.618,1.032)	0.56	0.617	78.4	-11.9
HBsAg level	11	-0.023(-0.465,0.419)	0.195	0.909	84.3	-13.7
HBcAb level	12	0.118(-0.177,0.413)	0.132	0.394	84.2	-4.27
Publication year	15	-0.013(-0.126,0.099)	0.052	0.8	86.8	-6.34
**Endpoint type (Ref: Relapse)**	15			(p = 0.739)	84.9	-15.1
HBVr		0.108(-1.154,1.369)	0.566	0.853		
VBT		0.508(-1.161,2.177)	0.749	0.513		
Acute exacerbation		-0.141(-1.249,0.967)	0.497	0.783		
Other		0.644(-0.629,1.916)	0.571	0.286		
**Cut-off value (Ref: ≥4 log_{10} U/mL)**	15			(p = 0.854)	85	-26.8
3-4log_{10}IU/mL		-0.503(-1.526,0.520)	0.459	0.299		
<3log_{10}IU/mL		-0.388(-2.141,1.365)	0.787	0.632		
N/A		-0.382(-1.706,0.942)	0.594	0.535		
≥0.275OD		-0.227(-1.933,1.480)	0.766	0.773		
**Detection time (Ref: Postoperative day 3)**	15			(p = 0.412)	81.8	2.31
EOT		-0.502 (-2.115, 1.112)	0.713	0.5		
Pre-chemotherapy		-0.529 (-2.419, 1.361)	0.836	0.542		
EOT 12w		-0.109 (-2.635, 2.417)	1.117	0.924		
Postpartum W12 HBcrAg		-0.483 (-2.634, 1.667)	0.951	0.623		
Baseline		-1.486 (-3.623, 0.650)	0.944	0.15		

Variable	No. of Studies	β(95%CI)	SE	p-value	Residual Heterogeneity (I²_res, %)	Adjusted R² (%)
**Study quality (Ref: Moderate risk)**				(p = 0.288)	82.2	6.67
High risk		-1.044 (-2.466, 0.378)	0.652	0.136		
Low risk		-0.014 (-0.790, 0.761)	0.356	0.968		
**Study design (Ref: Retrospective)**	15			(p = 0.629)	86.8	-8.07
RCT		0.188(-1.494,1.869)	0.772	0.812		
Prospective		-0.328(-1.258,0.602)	0.427	0.457		

Age: Represented by the median or mean age of participants in each study.HBsAg Level: Baseline HBsAg values (median or mean) from included studies, measured in log IU/mL.HBcrAg Level: Baseline HBcrAg values (median or mean), reported in log U/mL.
