# Supplementary figures and images for: Predictive Value of Hepatitis B Core-Related Antigen for Multiple Recurrence Outcomes After Treatment Cessation in Chronic Hepatitis B: A Meta-Analysis Study
Source: Viruses. 2025 Jun 30;17(7):929. doi: 10.3390/v17070929 (PMC12299212; doi:10.3390/v17070929)

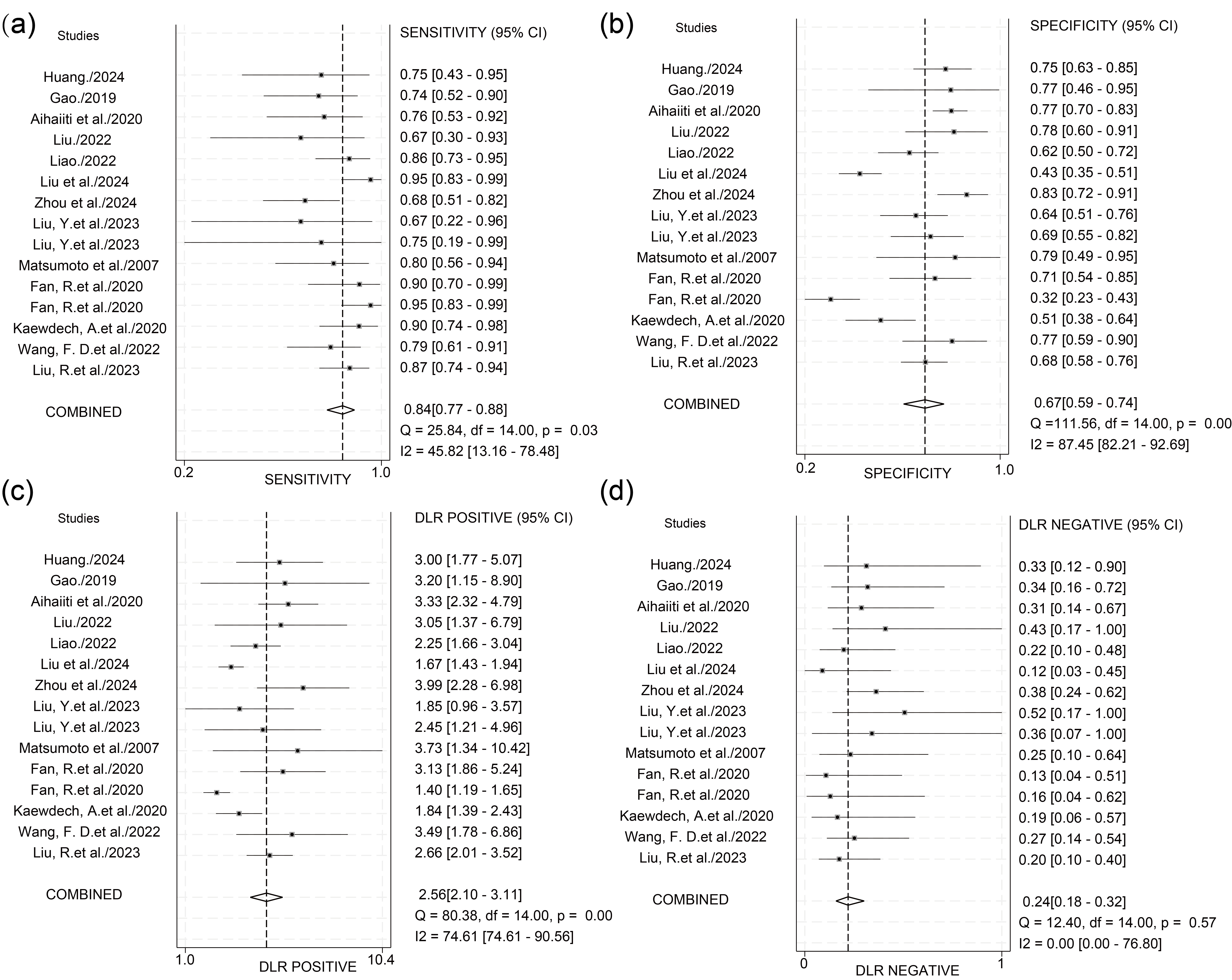

Supplement: Supplementary file 1 [file viruses-17-00929-s001.zip › FS1.jpg]

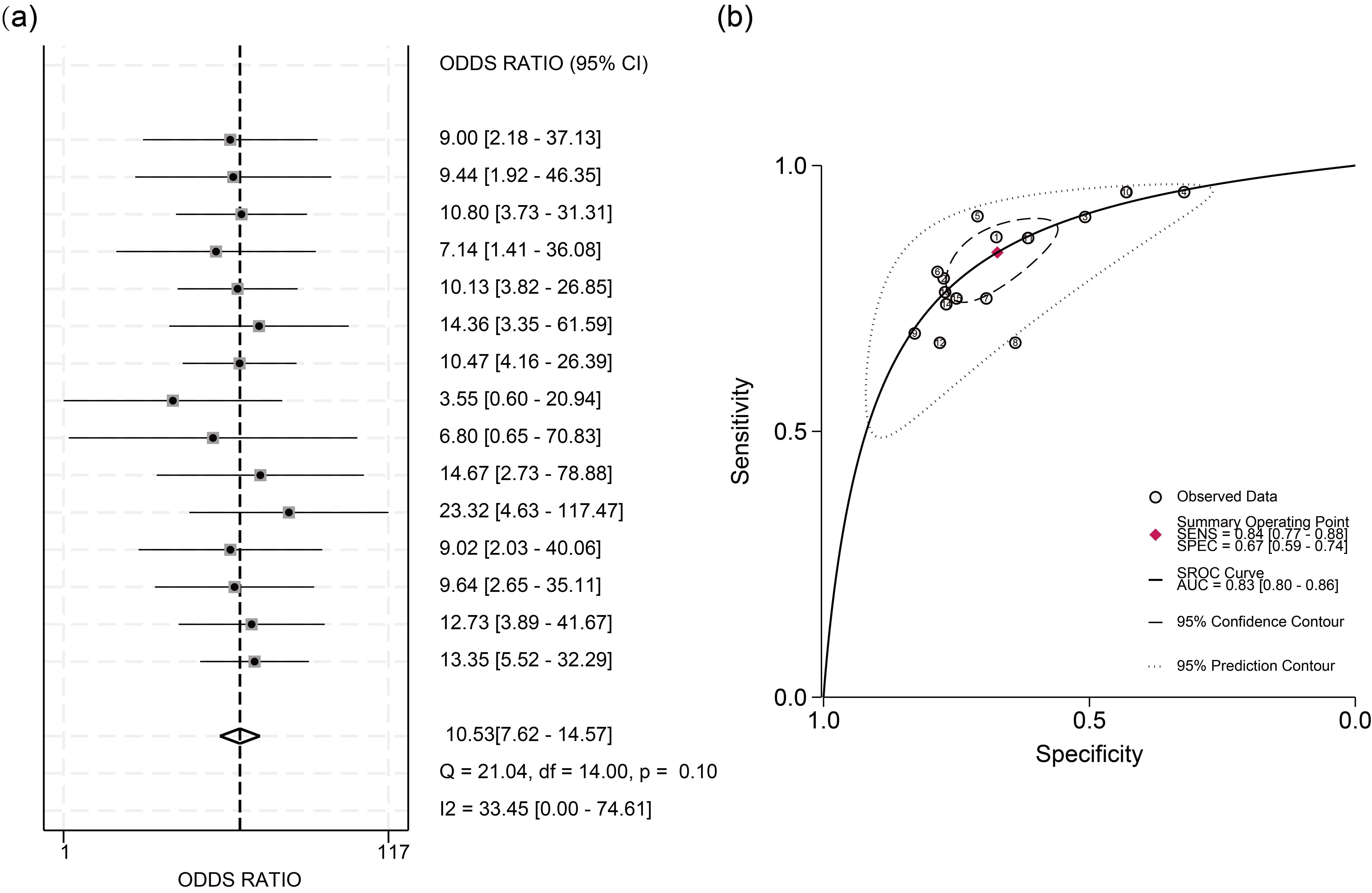

Supplement: Supplementary file 1 [file viruses-17-00929-s001.zip › FS2.jpg]
